# Structural Modeling Insights into Human VKORC1 Phenotypes

**DOI:** 10.3390/nu7085313

**Published:** 2015-08-14

**Authors:** Katrin J. Czogalla, Matthias Watzka, Johannes Oldenburg

**Affiliations:** 1 Institute of Experimental Hematology and Transfusion Medicine, University Clinic Bonn, Bonn 53105, Germany; E-Mails: katrin.czogalla@ukb.uni-bonn.de (K.J.C.); matthias.watzka@ukb.uni-bonn.de (M.W.); 2 Center for Rare Diseases Bonn (ZSEB), University Clinic Bonn, Bonn 53127, Germany

**Keywords:** vitamin K epoxide reductase (VKOR), VKORC1, vitamin K, vitamin K 2,3-epoxide, warfarin, VKCFD2, molecular modeling, vitamin K antagonists

## Abstract

Vitamin K 2,3-epoxide reductase complex subunit 1 (VKORC1) catalyses the reduction of vitamin K and its 2,3-epoxide essential to sustain γ-carboxylation of vitamin K-dependent proteins. Two different phenotypes are associated with mutations in human VKORC1. The majority of mutations cause resistance to 4-hydroxycoumarin- and indandione-based vitamin K antagonists (VKA) used in the prevention and therapy of thromboembolism. Patients with these mutations require greater doses of VKA for stable anticoagulation than patients without mutations. The second phenotype, a very rare autosomal-recessive bleeding disorder caused by combined deficiency of vitamin K dependent clotting factors type 2 (VKCFD2) arises from a homozygous Arg98Trp mutation. The bleeding phenotype can be corrected by vitamin K administration. Here, we summarize published experimental data and *in silico* modeling results in order to rationalize the mechanisms of VKA resistance and VKCFD2.

## 1. Introduction

Vitamin K 2,3-epoxide reductase complex subunit 1 (VKORC1) is the rate limiting enzyme of the vitamin K cycle [1,2]. VKORC1 is located in the endoplasmic reticulum (ER) membrane and catalyses reduction of vitamin K 2,3-epoxide (K > O) to vitamin K quinone (K) and further to vitamin K hydroquinone (KH_2_) [3]. Vitamin K reduction to KH_2_ is essential for the enzyme γ-glutamyl carboxylase (GGCX) to modify vitamin K-dependent (VKD) proteins at their Gla domains. During γ-carboxylation, KH_2_ is oxidized to K > O. The K > O is reduced to vitamin K quinone and further to KH_2_ by VKORC1, completing a recycling mechanism known as the vitamin K cycle [4]. A functional vitamin K cycle including VKORC1 and GGCX is required to enable physiological function of all VKD proteins. VKD proteins are involved in blood coagulation (coagulation factors FII, FVII, FIX, FX, Protein C, S, and Z) as well as in calcium homeostasis (matrix Gla protein, MGP; bone Gla protein, OST) [5,6]. There have been several additional VKD proteins reported to be involved in other pathways including cell-cycle regulation or with unknown functions [7].

Since the *VKORC1* gene was cloned in 2004, genetic analysis has revealed a number of mutations causing one of two different pathological phenotypes [8,9,10]. To date, 28 human *VKORC1* mutations have been identified that cause resistance to several vitamin K antagonists (VKA) that are used clinically as oral anticoagulants [11,12]. Patients with these mutations require higher VKA doses for stable anticoagulation. Other 4-hydroxycoumarin and indandione derivatives are used as rodenticides. Historically, warfarin has been the most common VKA used in the anticoagulant clinic or as a rodenticide, and it has been common terminology to describe patients and animals with resistance to any VKA as possessing “warfarin resistance” (WR). This convention will be used in this manuscript. Interestingly, there are also several rat and mouse VKORC1 mutations reported to cause WR that affect residues homologous to known warfarin resistance mutations in human VKORC1 (hVKORC1) [13,14].

In contrast, there is only one mutation known to result in the VKCFD2 phenotype. VKORC1:p.Arg98Trp causes diminished vitamin K epoxide reductase (VKOR) activity compared to that of the wild-type enzyme [15]. VKCFD2 patients exhibit severely diminished activities for the VKD coagulation factors and suffer spontaneous or surgery/injury induced bleeding episodes [16,17]. In addition to this haemorrhagic phenotype, abnormalities in epiphyseal growth have been reported in one case [18]. This phenotype is very rare. Worldwide, there are only four unrelated families known to be affected with VKCFD2 [16,17,18].

This review discusses features of the modeled structure of the human VKORC1 enzyme, putative amino acid sequences involved in warfarin binding, and motifs that influence ER-retention of the enzyme and proposed general mechanisms that can explain the respective phenotypes.

## 2. The Crystal Structure of *Synechococcus* VKOR—A Homolog to hVKORC1

The first X-ray crystallographic structure of *Synechococcus* sp. VKOR (synVKOR), a bacterial homolog of the hVKORC1 enzyme, was reported by Li *et al.* in 2010 [19]. This enzyme is composed of five transmembrane helices (TMs). The first four TMs form a bundle surrounding a quinone in its interior thereby comprising the catalytic core of synVKOR. The quinone substrate is close to the periplasmic side of the enzyme and in close proximity to the CXXC active site motif located in TM4. There is a long periplasmic loop between TM1 and TM2 that includes a ½ helix (“½ segment” in the original article) and a pair of cysteines (Cys50, Cys56) and a serine/threonine residue (Ser62) conserved among all VKOR homologs [20]. The fifth TM of synVKOR is located outside of the four-helix bundle and is connected via a C-terminal linker segment to a thioredoxin (Trx)-like domain. The Trx-like domain is the naturally fused redox partner of synVKOR. Li *et al.* [19] observed a disulfide bridge with strong electron density between Cys50 in the periplasmic loop of the VKOR domain and Cys209 of the Trx-like domain. Thus, the synVKOR structure suggests an electron transfer mechanism that shuttles reducing equivalents from the Trx-like domain, via the conserved cysteines in the loop, to the CXXC active site motif where the bound ubiquinone substrate becomes reduced to the hydroquinone form [21].

## 3. The Human VKORC1 Homology Model

In the absence of high-resolution X-ray crystallographic or NMR structures of hVKORC1, creation of a homology model based on the synVKOR structure provides an opportunity to gain structure-based insights into hVKORC1 function. Several *in silico* algorithms revealed conflicting topology predictions of either a 3TM, 4TM or 5TM structure for *hVKORC1* [8,9,11,22,23,24]. There are also conflicting experimental data supporting 3TM or 4TM topologies [25,26,27]. Nevertheless, all VKOR homologues share conserved functional residues at homologous positions which strongly suggest that all homologs share a common protein fold and topology with respect to the lipid membrane in which they are embedded.

## 4. Conserved Amino Acid Residues of Human VKORC1

VKORC1 homologues are found in plants, bacteria, archaea and mammals but not in yeast and fungi [20,28,29]. All VKOR homolog enzymes possess a CXXC motif in the active site, essential for reduction of quinone substrates [3,20,30,31,32]. The cysteines of the CXXC motif form a disulfide bridge that becomes reduced for catalytic activity. There are two additional cysteines (Cys43 and Cys51) that can form a disulfide bond and a conserved serine at amino acid position 57 (or either threonine in some VKORC1 homologues) in the large loop of hVKORC1 [3,19,30,32,33]. These four cysteines and the serine/threonine are absolutely conserved and define proteins of the VKOR family.

**Figure 1:**
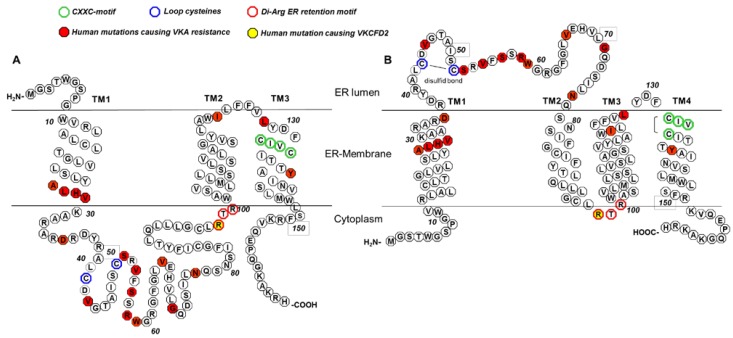
3TM and 4TM topological models for hVKORC1 (modified from Tie *et al.*, 2012 [26]). In both models, conserved cysteines (Cys132 and Cys135 of the active center (CXXC motif), green; loop cysteines Cys43 and Cys51, blue) and Arg98_Arg100 of the di-arginine endoplasmic reticulum (ER) retention motif (red) are labeled with colored circles. Amino acid positions for which mutations were reported to be associated with either vitamin K antagonist (VKA) resistance or combined deficiency of vitamin K dependent clotting factors type 2 (VKCFD2) are marked by filled circles (mutations causing VKA resistance, red; VKCFD2 mutation, yellow). (**A**) Shows the putative topology for hVKORC1 as a 3 TM membrane-embedded protein with the loop located in the cytoplasm. The N-terminus is located in the ER lumen, whereas the C-terminus is in the cytoplasm. (**B**) Shows the putative 4TM topology for hVKORC1 with the loop containing the conserved cysteines Cys43 and Cys51 in the ER lumen with both termini located in the cytoplasm.

The CXXC motif is located in the last TM and is oriented towards the ER lumen in both 3TM and 4TM models for hVKORC1 (Figure 1). All published reports show complete loss of VKOR activity if one of the cysteines in the CXXC motif is mutated (Cys132 and Cys135, Table 1) [3,19,30,31]. These data clearly demonstrate that the CXXC motif is the active center essential for substrate reduction. The functional role of the conserved cysteines in the large loop, Cys43 and Cys51, is not completely clear. Due to their localisation Cys43 and Cys51 are also called “loop cysteines”. Of the two topology models, studies proposing the 4TM model claim that the loop cysteines are essential for *in vivo* VKOR activity [27,33], whereas studies supporting the 3TM model argue that they are not necessary [3,26,34]. The main difference between both models is the orientation of the N-terminus, the first TM, and the large loop. In the 3TM model, the loop is located in the cytoplasm, whereas in the 4TM model, the loop is in the ER lumen (Figure 1). In the 4TM model, the loop cysteines are required for electron transfer from redox partners located in the ER lumen to reduce the CXXC motif. This postulated electron transfer pathway is necessary for catalytic VKOR activity and is also present in bacterial VKOR homologues. In synVKOR, the disulfide bridge formed between the loop cysteines is reduced by a periplasmic Trx-like redox partner naturally fused to the VKOR core protein. In vertebrates, this natural fused redox partner is missing. Therefore, Trx-like domain containing proteins are thought to reduce the loop cysteines in vertebrates. Candidate partner oxidoreductases include protein disulfide isomerases (PDIs) that are either separate globular proteins in the ER lumen or type-I ER membrane-anchored proteins with redox-active cysteines facing the ER lumen [27,35]. Thus, electron transfer mediated by the loop cysteines is feasible for the 4TM model only, as most PDIs are resident in the ER lumen. However, for the 3TM model, PDIs would not be able to reduce the loop cysteines if the loop is located in the cytoplasm. Here, the two loop cysteines could not be essential for VKOR activity, and electron transfer would presumably take place through direct reduction of the CXXC motif. Thus, location of the loop cysteines in the ER lumen might not be a strict requirement for hVKORC1 function if the 3TM model were to be correct.

**Table 1:** Published activities for hVKORC1 conserved cysteines by various functional assays.

Publication	Rost *et al.* [30]	Jin *et al.* [3]	Rishavy *et al.* [33]	Tie *et al.* [26]	Tie *et al.* [34]	Tie *et al.* [36]
**Type of VKOR assay**	DTT-driven assay	DTT-driven assay	DTT-driven assay	Cell-based assay	DTT-driven assay/cell-based assay	Cell-based assay
**Reductant**	DTT	DTT	DTT/Trx/TrxR	-	-	-
**Cell line**	HEK293 cells	Sf9 cells	Sf21 cells	HEK293 cells	HEK293 cells	C1 + L1 DKO HEK cells
**Cysteine residue variants:**						
**Cys43Ala**	20%	25%	~85%/0%	<5%	25%/<5%	<5%
**Cys43Ser**	20%					
**Cys51Ala**		100%	~50%/0%	95%	100%/100%	105%
**Cys51Ser**	<5%					
**Cys43Ala + Cys51Ala**		112%		60%	85%/110%	90%
**Cys43_Cys51del**		85%			85%/60%	
**Cys132Ala/Ser**	<5%	0%			0%/0%	
**Cys135Ala/Ser**	<5%	0%			0%/0%	

This table shows the published vitamin K epoxide reductase (VKOR) activities of human vitamin K 2,3-epoxide reductase complex subunit 1 (hVKORC1) variants in which conserved cysteines were selectively mutated or deleted. In each published study the VKOR activities are presented as a percentage of the activity of the wild-type hVKORC1 reported in the same study. The table also lists the type of VKOR assay, reductant (dithiothreitol (DTT) or thioredoxin/thioredoxin reductase (Trx/TrxR)), and cell line (human embryonic kidney (HEK) cells, *Spodoptera frugiperda* (Sf21) cells, or double knock-out (DKO) HEK cells) used.

It follows from the arguments presented above that VKOR activity measurements of variants affecting the loop cysteines might provide important clues to elucidate the topology and structure of hVKORC1. Reduced activities for amino acid substitutions of loop cysteines would tend to support the 4TM model where loop cysteines would be essential for electron transfer. Alternatively, data revealing unaffected activity for these variants would favour the 3TM topology model where loop cysteines would not be required for CXXC motif reduction. Several research groups have published conflicting activity data regarding the loop cysteines supporting either the 3TM or 4TM topology (Table 1). Rost *et al.* [30] were the first to publish data regarding the loop cysteines of hVKORC1 investigated by the commonly used dithiothreitol (DTT)-driven VKOR assay. They demonstrated a reduction of VKOR activity to 5% for Cys51Ser and to 20% for Cys43Ser with respect to wild-type activity [30]. By contrast, Jin *et al.* published results revealing more than 100% activity for Cys51Ala compared to that of wild-type VKORC1. However, the Cys43Ala variant showed reduced activity to 25% which corresponds well to the data of Rost *et al.* for Cys43Ser. Interestingly, VKOR activity of a variant with both loop cysteines mutated was unaffected (Table 1) [3]. Rishavy *et al.* [33] modified this *in vitro* VKOR assay by replacing the non-physiological DTT with the reductant thioredoxin/thioredoxin reductase (Trx/TrxR). In their study, they demonstrated that VKOR activity assessed by the DTT-driven assay is prone to false positive results. They showed that membrane-permeable DTT directly reduces the active centre of hVKORC1, essentially bypassing the loop cysteines. Thus, the loop cysteine variants showed VKOR activity when DTT was used as reductant. When DTT was replaced by the membrane impermeable Trx/TrxR reductant, VKORC1 loop cysteine mutants did not show VKOR activity (Table 1) [33]. This strongly suggests that loop cysteines are essential for VKOR activity when driven by a biological oxidoreductase enzyme. However, the study was negatively critiqued regarding the relative concentration differences between DTT and Trx/TrxR concentrations used (millimolar for DTT *vs.* micromolar for Trx/TrxR) [22]. Alternatively, loop cysteine variants have been examined in one of the recently reported cell-based assays in which no detergent and extra reductants are used and VKOR activity is indirectly assessed in living cells. Two studies by Tie *et al.* [26,36] revealed very low VKOR activity for Cys43Ala (<5%), whereas Cys51Ala showed activity comparable to that of wild-type VKORC1. Again, they found that mutation of both loop cysteines did not affect VKOR activity (Table 1) [26,36].

Taken together, data from all research groups demonstrated reduction of VKOR activity for all variants affecting Cys43, independent of the type of assay used. However, results regarding activities of Cys51 variants are in disaccord as they range between 0% and 100% relative to wild-type VKOR activity. Moreover, these data do not unequivocally clarify whether or not the loop cysteines are essential for hVKORC1 activity and, thus, do not definitively support either the 3TM or 4TM model.

## 5. The 3TM VKORC1 Topology Model

The 3TM model is characterized by three transmembrane alpha-helices with a large cytoplasmic loop containing the conserved loop cysteines located between the first and second TMs. The C-terminus is located in the cytoplasm and the N-terminus in the ER lumen (Figure 1A). The 3TM model is supported by several *in silico* topology prediction algorithms and by experimental data from a fluorescence protease protection (FFP) assay of Tie *et al.* [26,37]. In this assay, VKORC1 enzyme was either C- or N-terminal tagged with the green fluorescent protein (GFP). After expression of both GFP tagged variants in HEK293 cells, a GFP digestion was performed using trypsin. The cells were treated before the digestion with digitonin to selectively permeabilize only the plasma membrane so that GFP tags located in the cytoplasm, but not in the ER lumen, become digested by the protease. The FFP assay results revealed a complete digestion of GFP for the C-terminal tagged VKORC1 protein after 90 s, whereas the N-terminal GFP-tagged VKORC1 continued to show a fluorescent signal even after 150 s. These data suggest that N- and C-termini are located on opposite sides of the ER membrane with the C-terminus in the cytoplasm. The N-terminus appears to be located in the ER lumen because it is protected from the proteolysis [26].

## 6. The 4TM VKORC1 Topology Model

The putative 4TM topology for hVKORC1 is composed of 4TM domains and includes the large ER lumenal loop between TM1 and TM2 with the two conserved loop cysteines. Both termini are located in the cytoplasm (Figure 1B). The CXXC motif is located in 4TM close to the ER lumen, equivalent to its placement in the third and final TM for the 3TM model. The crystal structure of the bacterial synVKOR enzyme strongly suggests a 4TM topology for hVKORC1. The membrane embedded four-helix bundle of synVKOR is homologous to hVKORC1 which shares ~24% primary sequence identity [19].

Wajih *et al.* [35] performed a knock-down of PDI expression by using siRNA silencing. This revealed reduced VKOR activity to 25% in HEK293 cells. In addition, they demonstrated by sodium dodecyl sulfate polyacrylamide gel electrophoresis (SDS-PAGE) that PDIs form a complex with VKORC1 [35]. This was the first convincing evidence that PDIs may be physiological redox partners for hVKORC1. Further immunoprecipitation data by Li *et al.* [27] confirmed which PDI family member interacts with hVKORC1 [27]. The interaction was only present if Cys51 of hVKORC1 and the second active site cysteine of the Trx-like domain of PDI were both mutated to alanine. These amino acid substitutions were necessary because the native cysteine pairs of VKORC1 and PDI are self-reducing during redox cycling and do not form stable disulfide bonds. As a result of the mutagenesis, the “donor” cysteine of PDI and the “acceptor” cysteine (Cys43) of hVKORC1 form a stable disulfide bond that remains oxidized during immunoprecipitation and detection. These results indicate that at least three PDIs identified can transfer electrons to the loop cysteines of hVKORC1. *In silico* modeling of hVKORC1 on the X-ray crystallographic structure of synVKOR also suggests a 4TM topology [19,23]. Additionally, *in silico* docking of warfarin on this model disclosed three putative binding interfaces. All human, as well as rodent, VKORC1 mutations associated with warfarin resistance are located in or close to these putative binding interfaces, supporting the plausibility of our *in silico* analysis (see detailed information in the next section) [23].

Taken together, data from the PDI knock-down, immunoprecipitation interaction studies, and *in silico* modeling of hVKORC1 with the identification of putative warfarin binding interfaces are largely consistent with a 4TM topology for hVKORC1 (Table 2).

**Table 2:** Overview of published experimental data supporting 3TM or 4TM topological models for hVKORC1.

Arguments for 3TM hVKORC1 Structure	Arguments for 4TM hVKORC1 Structure
Location of the C-Terminus of VKORC1 in the cytoplasm and of the N-Terminus in the ER-lumen; FFP assay [26]	siRNA knock-down of PDI located in the ER lumen results in reduced VKOR activity [35]
Cys51Ala exhibits VKOR activity = Cys51 is not required for VKOR activity, DTT and cell-based assays [3,26,36]	Cys43Ala/Ser and Cys51Ala/Ser exhibit no VKOR activity = Cys43 and Cys51 are required for VKOR activity, DTT and Trx/TrxR assays [30,33]
hVKOR model, prediction program TOPCONS [37]	Cys43 forms a disulfide bond with four PDIs, immunoprecipitation [27]
	3.6 Å crystal structure of the bacterial homologue of VKOR from *Synechococcus* sp. in conjunction with multiple sequence alignments [19]
	hVKORC1 model based on crystal structure of synVKOR and putative warfarin binding interfaces that correspond to the reported WR mutations [23]

The left- and right-hand columns present brief summaries of the experimental data obtained from structural studies and assays of VKOR activity (together with literature citations) that support either the 3TM or 4TM topology for hVKORC1 respectively.

## 7. Warfarin Binding and Mutations Causing Warfarin Resistance

VKA such as warfarin, are direct inhibitors of VKORC1 and are used in the prevention and therapy of thromboembolic disorders [4,29]. It is widely believed that warfarin binds to VKORC1 irreversibly [38]. To date, genetic analysis of *VKORC1* has implicated a total of 28 mutations to be associated with WR in humans (Table 3) [8,11,12]. Affected patients require greater doses of VKA drugs for stable oral anticoagulation compared to wild-type VKORC1 probands. Some WR patients even exhibit an apparent complete resistance leading to ineffective control or to the abandonment of anticoagulation therapy with VKA [11,12].

**Table 3:** Comparison of warfarin inhibition for hVKORC1 variants determined by *in vitro* assays of vitamin K 2,3-epoxide reductase (VKOR) in cell fractions or in cultured cells.

hVKORC1 Variant	Mean Patient Dosage in HDT Multiples [Drug] for *n* = Number of Reported Patients [11]	Warfarin IC_50_ by DTT-Driven VKOR Assay [8,12]	Warfarin IC_50_ by Cell Based Assay [23]	Warfarin Phenotypes by Cell Based Assay [36]
Wild-type	1.0 [W, P] (*n* = 77)			
Ala26Pro	>3.0 [W] (*n* = 1)	11.2-fold increased K_i_[12]	49.6-fold increased IC_50_	n.d.
Ala26Thr	>2.0 [P] (*n* = 1)	sensitive as wt [12]	3.0-fold increased IC_50_	n.d.
Leu27Val	>3.0 [F], 1.0 [W] (*n* = 1) *	sensitive as wt [12]	2.5-fold increased IC_50_	n.d.
His28Gln	3.5 [P] (*n* = 1)	more sensitive than wt [ 12]	2.9-fold increased IC_50_	n.d.
Val29Leu	2.0 [W] (*n* = 1)	absence of expression [12]/low VKOR activity and more sensitive than wt [8]	5.5-fold increased IC_50_	n.d.
Ala34Pro	3.8 [W] (*n* = 1)	n.d.	n.d.	n.d.
Asp36Gly	3.0 [W] (*n* = 1)	more sensitive than wt [ 12]	3.2-fold increased IC_50_	n.d.
Asp36Tyr	1.5–3.5 [W] (*n* = 10)	sensitive as wt [12]	3.8-fold increased IC_50_	n.d.
Val45Ala	>2.0 [W] (*n* = 1)	low VKOR activity [ 8], more sensitive than wt [8,12]	6.2-fold increased IC_50_	n.d.
Ser52Leu	>3.0 [P] (*n* = 1)	low VKOR activity, K_i_ determination not possible [ 12]	7.4-fold increased IC_50_	moderate resistance
Ser52Trp	3.5 [P] (*n* = 1)	low VKOR activity, K_i_ determination not possible [ 12]	5.7-fold increased IC_50_	sensitive as wt
Val54Leu	1.5–5.5 [W] (*n* = 2)	4.6-fold increased K_i_[12]	4.5-fold increased IC_50_	n.d.
Ser56Phe	>5.0 [P] (*n* = 1)	more sensitive than wt [12]	6.8-fold increased IC_50_	n.d.
Arg58Gly	5.0 [W] (*n* = 1)	low VKOR activity [8], more sensitive than wt [8 and 12]	3.4-fold increased IC_50_	n.d.
Trp59Arg	7.0 [P] (*n* = 1)	low VKOR activity, K_i_ determination not possible [12]	17.5-fold increased IC_50_	high resistance
Trp59Cys	>3.5 [P] (*n* = 1)	more sensitive than wt [12]	7.6-fold increased IC_50_	n.d.
Trp59Leu	>5.0 [P] (*n* = 1)	low VKOR activity, K_i_ determination not possible [12]	75.2-fold increased IC_50_	high VKOR activity, high resistance
Val66Gly	2.5 [P] (*n* = 1)	low VKOR activity, K_i_ determination not possible [12]	2.8-fold increased IC_50_	sensitive as wt
Val66Met	3.0–6.0 [W] (*n* = 7)	low VKOR activity, K_i_ determination not possible [12]	5.4-fold increased IC_50_	sensitive as wt
Gly71Ala	>2.0 [P] (*n* = 1)	low VKOR activity, K_i_ determination not possible [12]	5.1-fold increased IC_50_	sensitive as wt
Asn77Ser	>3.0 [P] (*n* = 1)	low VKOR activity, K_i_ determination not possible [12]	5.3-fold increased IC_50_	moderate resistance
Asn77Tyr	3.5 [W] (*n* = 1)	low VKOR activity, K_i_ determination not possible [12]	3.9-fold increased IC_50_	sensitive as wt
Ile123Asn	>7.0 [P] (*n* = 1)	2.4-fold increased K_i_[12]	8.5-fold increased IC_50_	n.d.
Leu128Arg	>4.0–7.0 [W] (*n* = 5)	low VKOR activity [8,12], K_i_ determination not possible [12]/more sensitive than wt [8]	49.7-fold increased IC_50_	high VKOR activity, high resistance
Tyr139His	>3.0 [W] (*n* = 1)	3.6-fold increased K_i_[12]	4.6-fold increased IC_50_	n.d.

This table shows patient data as well as *in vitro* results from the DTT-driven and cell culture-based VKOR assays for VKORC1 variants reported to cause resistance to VKA. Patient data from Watzka *et al.* [11] and Hodroge *et al.* [12], with [P] = phenprocoumon, [W] = warfarin; HDT = High Dosage Threshold which is equivalent to the mean patient population dosage divided by that for the control group (homozygous wild-type *VKORC1* alleles with *VKORC1*:c.-1639GG haplotype). The patient marked with an asterisk (*) and Leu27Val mutation had additionally the CYP2C9*2*3 haplotype which results in a reduced warfarin dosage requirement to achieve a stable, therapeutic INR compared to patients with wild-type CYP2C9*1*1 haplotype. Variants investigated by the DTT-driven assay by Hodroge *et al.* [12] and Rost *et al.* [8] were summarized in one column, followed by data from Czogalla *et al.* [23] and Tie *et al.* [36]. n.d. = not determined.

Investigation by *in vitro* assays of these mutations is challenging and the results often do not correlate with patient phenotypes [39]. Rost *et al.* (2004) first investigated warfarin inhibition of six WR variants heterologously expressed in HEK293 cells using the DTT-driven VKOR assay but none of these mutations showed an *in vitro* resistance phenotype [8]. Similarly, Hodroge *et al.* (2012) expressed human WR variants in *Pichia pastoris* and found that only four of 25 mutations investigated revealed resistance phenotypes by the DTT-driven VKOR assay [12]. Nineteen variants were either non-functional or did not show a resistance phenotype [12]. Ten of the mutations that had essentially no *in vitro* VKOR activity in the study of Hodroge *et al.* (2012) [12] were re-examined by Tie *et al.* (2013) [36] using a cell-based assay. This assay indirectly reports activity of hVKORC1 using carboxylation of a coexpressed chimeric VKD protein in a HEK293 cell line constitutively knocked-out for both endogenous *VKORC1* and its paralog enzyme *VKORC1L1* [36]. For all the variants investigated, Tie *et al.* [36] detected VKOR activity as great as or greater than that of wild-type VKORC1. Five mutations exhibited a warfarin resistance phenotype by their cell culture-based assay, consistent with reported patient WR phenotypes [35]. However, five other WR mutations were not resistant to warfarin in their assay [36]. Hodroge *et al.* (2012) [12] and Tie *et al.* (2013) [36] both speculated that therapeutic doses of warfarin in these patients possibly have impact alternative enzyme targets other than the hVKORC1 mutated variants. In contrast, our group showed that all known hVKORC1 mutations associated with WR revealed *in vitro* WR phenotypes by a similar cell culture-based assay [23,39]. In all cases, we measured *in vitro* WR phenotypes ranging from mild to total resistance, in agreement with elevated patient dosages previously reported (Table 3) [11]. Discrepancies detected for WR phenotypes by the cell culture-based assays of Tie *et al.* [36] and of Czogalla *et al.* [23,39] might be explained by differences in the experimental setting. The genomic background of the HEK293T cell strains used was a wild-type in the study of Czogalla *et al.* [23] and a *VKORC1/VKORC1L1* double knock-out in the study of Tie *et al.* [36]. Additionally, our group [23,39] used wild-type FIX as a coexpressed VKD reporter protein to measure VKORC1 function, whereas Tie and co-workers [36] used a chimeric construct comprising protein C with its Gla domain replaced by that of FIX. Similar differences in the ability of DTT-based and cell culture-based assays to report *in vitro* WR phenotypes for mouse and rat VKORC1 have been reported [13,14,40].

To gain further insight into the binding and inhibitory action of warfarin, we performed *in silico* docking of warfarin on our model of hVKORC1. Molecular docking analysis revealed three putative warfarin binding interfaces on hVKORC1 comprising linear sequences of the ER-lumenal loop (Ser52-Phe55), and the first (Leu22-Lys30) and fourth (Phe131-Thr137) transmembrane helices of hVKORC1 [23]. Twenty-four human and nine rodent VKORC1 mutations confirmed to be WR are located at or near to the predicted binding interfaces. In addition, all amino acid substitutions causing WR, except for Tyr139His, were found to have their side chains on the ER lumenal side of the enzyme, thereby potentially influencing warfarin binding. Furthermore, our data suggest that warfarin might inhibit hVKORC1 in three possible ways. Firstly, binding of warfarin may interfere with the redox cycling of the disulfide bridge between the loop cysteines (Cys43 and Cys51). Secondly, warfarin binding might hinder the close approach of the reduced loop cysteines to the active centre of VKORC1 (CXXC motif). Thirdly, binding of warfarin might also hinder access of the K > O substrate to its binding pocket in the hVKORC1 active site. In summary, the insight gained through comparative modeling of hVKORC1 based on the synVKOR X-ray crystallographic structure has led to further investigation of the molecular mechanisms of warfarin inhibition of hVKORC1 and warfarin resistance.

## 8. VKCFD2

To date, there have been only three unrelated families reported with probands suffering from VKCFD2 who harbour homozygous VKORC1:p.Arg98Trp alleles [16,17]. Instead of WR, this mutation causes spontaneous bleedings due to reduced VKOR activity of 20%–60% compared to that for homozygous wild-type hVKORC1 individuals [8,16,17,18]. A previous study confirmed diminished, warfarin-sensitive VKOR activity of ~10%, relative to that of wild-type enzyme by the DTT-driven VKOR assay [30]. However, the haemorrhagic phenotype of these patients can be corrected by vitamin K administration which results in restoration of activities for the vitamin K-dependent clotting factors to normal ranges. Until recently, the pathophysiological mechanism underlying the single mutation causing VKCFD2 was unknown.

In a recent study, we modeled hVKORC1 and performed *in silico* analysis of the region of interest. We found that the Arg98Trp mutation might be part of a putative di-arginine ER retention motif [15]. To confirm these *in silico* results experimentally, different variants affecting Arg98 and/or Arg100 of the putative ER retention motif were expressed in HEK293T cells and analysed by fluorescence confocal microscopy. As expected, wild-type hVKORC1 was exclusively located in the ER, whereas all variants affecting the di-arginine ER retention motif revealed reduced ER localization to only 9% compared to that of wild-type hVKORC1. The naturally occurring variant Arg98Trp resulted in 20% ER-localization compared to that of the wild-type. We further observed that the protein variants affecting the di-arginine ER retention motif were directed to the cytoplasm. If the residual amount of hVKORC1:Arg98Trp in the ER is not functionally impaired with respect to VKOR activity, this would explain phenotype correction by vitamin K administration for VKCFD2 patients [41].

Interestingly, our identified di-arginine ER retention motif (Arg98_Arg100) is consistent with both the 3TM and 4TM topology models for hVKORC1. Di-arginine based ER retention motifs are generally present in cytosolic domains of proteins at approximately 16–46 A° from the lipid bilayer and allows for interaction with ER retention proteins [42,43]. This is observed for both hVKORC1 models, in which the guanidyl groups of Arg98 and Arg100 are exposed to the cytoplasm. In the 4TM model, these arginines are located in the small cytoplasm-exposed loop connecting TM2 with TM3 (Figure 1B) [42,43]. However, our results would also support the alternative 3TM model for hVKORC1 where the di-arginine ER retention motif would be located at the end of the large putative cytoplasmic loop just before the TM2 helix (Figure 1A).

## 9. Conclusions

The identification of the human *VKORC1* gene in 2004 was a major step towards understanding the molecular and genetic mechanisms of the vitamin K cycle. Since then, 28 mutations causing WR and a single mutation responsible for VKCFD2 have been identified and functionally confirmed by *in vitro* studies. *In silico* analysis has provided insight into structural requirements for warfarin binding and the pathophysiological mechanism of VKCFD2.
